# Mono- and Binuclear Copper(II) and Nickel(II) Complexes with the 3,6-Bis(picolylamino)-1,2,4,5-Tetrazine Ligand

**DOI:** 10.3390/molecules26082122

**Published:** 2021-04-07

**Authors:** Oleh Stetsiuk, Abdelkrim El-Ghayoury, Francesc Lloret, Miguel Julve, Narcis Avarvari

**Affiliations:** 1 Univ Angers, CNRS, MOLTECH-Anjou, SFR MATRIX, F-49000 Angers, France; oleh.stetsiuk@polytechnique.edu (O.S.); abdelkrim.elghayoury@univ-angers.fr (A.E.-G.); 2 Departament de Química Inorgànica, Instituto de Ciencia Molecular (ICMol), Universitat de València, Catedrático José Beltrán 2, 46980 Paterna, Spain; Francisco.Lloret@uv.es

**Keywords:** 1,2,4,5-tetrazine, nitrogen ligands, nickel, copper, crystal structure determination, magnetic properties

## Abstract

Four new compounds of formulas [Cu(hfac)_2_(L)] (**1**), [Ni(hfac)_2_(L)] (**2**), [{Cu(hfac)_2_}_2_(µ-L)]·2CH_3_OH (**3**) and [{Ni(hfac)_2_}_2_(µ-L)]·2CH_3_CN (**4**) [Hhfac = hexafluoroacetylacetone and L = 3,6-bis(picolylamino)-1,2,4,5-tetrazine] have been prepared and their structures determined by X-ray diffraction on single crystals. Compounds **1** and **2** are isostructural mononuclear complexes where the metal ions [copper(II) (**1**) and nickel(II) (**2**)] are six-coordinated in distorted octahedral MN_2_O_4_ surroundings which are built by two bidentate hfac ligands plus another bidentate L molecule. This last ligand coordinates to the metal ions through the nitrogen atoms of the picolylamine fragment. Compounds **3** and **4** are centrosymmetric homodinuclear compounds where two bidentate hfac units are the bidentate capping ligands at each metal center and a bis-bidentate L molecule acts as a bridge. The values of the intramolecular metal···metal separation are 7.97 (**3**) and 7.82 Å (**4**). Static (dc) magnetic susceptibility measurements were carried out for polycrystalline samples **1**–**4** in the temperature range 1.9–300 K. Curie law behaviors were observed for **1** and **2**, the downturn of χ_M_*T* in the low temperature region for **2** being due to the zero-field splitting of the nickel(II) ion. Very weak [*J* = −0.247(2) cm^−1^] and relatively weak intramolecular antiferromagnetic interactions [*J* = −4.86(2) cm^−1^] occurred in **3** and **4**, respectively (the spin Hamiltonian being defined as ***H*** = −*J**S***_1_·***S***_2_). Simple symmetry considerations about the overlap between the magnetic orbitals across the extended bis-bidentate L bridge in **3** and **4** account for their magnetic properties.

## 1. Introduction

**Scheme 1:**
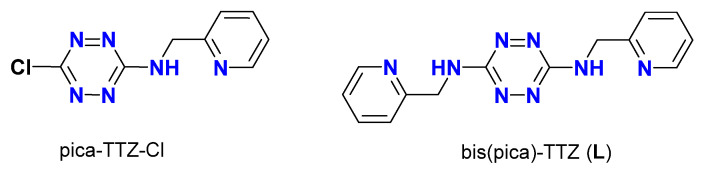
Picolylamine-1,2,4,5-tetrazine (TTZ) molecules; L was used as ligand in the present study.

The 1,2,4,5-tetrazine (TTZ) ring [1,2], thanks to its substituent dependent fluorescent properties [3,4,5,6], electron withdrawing character, allowing access to stable radical anion species [7,8,9], and active role in the establishment of anion-π interactions [10,11,12,13,14], has been considered as suitable platform for the attachment of coordinating groups towards multifunctional ligands and corresponding metal complexes [15,16]. Besides the possibility to access photoactive metal complexes [16], enhancement of the magnetic coupling between metal centers thanks to the bridging tetrazine radical anion can be achieved as well [17]. Accordingly, functionalization of TTZ in the positions 3 and 6 of the ring with nitrogen containing units allowed the preparation and use in various metal complexes of either ditopic and tetratopic ligands such as 3,6-(2′-dipyridyl)-TTZ [18,19], 3,6-dipyrimidyl-TTZ [20,21,22,23,24] and 3,6-(dipyrazolyl)-TTZ [25,26], or monotopic ligands, obtained from 3,6-dichloro-TTZ by nucleophilic substitution, such as dipicolylamine-TTZ-Cl [27], dipicolylamine-TTZ-tetrathiafulvalene [28] and dipicolylamine-TTZ-OMe [29]. Note that an interesting difference of the magnetic coupling between the metal centers in chloro-bridged dicobalt(II) and dimanganese(II) complexes with the dipicolylamine-TTZ-Cl ligand has been observed. Accordingly, antiferromagnetic coupling occurs in the latter, while the coupling is ferromagnetic in the cobalt(II) complex [27]. Although the magnetic interaction between the metal centers was not mediated by the tetrazine ring, these results highlight the opportunities arising from its use as a platform for functional ligands, affording complexes provided with interesting magnetic properties. Besides functionalization with dipicolylamine, the reaction of 3,6-dichloro-TTZ with 2-picolylamine (2-PyCH_2_NH_2_) in methyl-*t*-butylether provided either mono(picolylamine)-TTZ-Cl (pica-TTZ-Cl) [30] or bis(picolylamine)-TTZ (bis(pica)-TTZ L) [31] (Scheme 1) depending on the reaction time and temperature.

Pica-TTZ-Cl proved its versatile coordination behavior as a neutral or monoanionic ligand in a series of copper(II) mononuclear and mixed-valence copper(II)/copper(I) binuclear complexes [30], while with ligand L we have previously reported the cobalt(II) complexes of the formula [Co(hfac)_2_(L)]·CH_3_CN and {[Co(hfac)_2_(µ-L)][Co(hfac)_2_(CH_3_OH)_2_]}_n_ (hfac = hexafluoroacetylacetonate), which have shown field-induced slow magnetic relaxation, indicative of the so-called single-ion magnet (SIM) behavior [31].

In the continuation of our research on tetrazine-based ligands and complexes, we report herein the synthesis, in-depth structural characterization and magnetic properties of mono- and binuclear copper(II) and nickel(II) complexes with the bis(pica)-TTZ ligand L.

## 2. Results and Discussion

### 2.1. Synthesis and Characterization of TTZ-Based Complexes ***1**–**4***

**Scheme 2:**
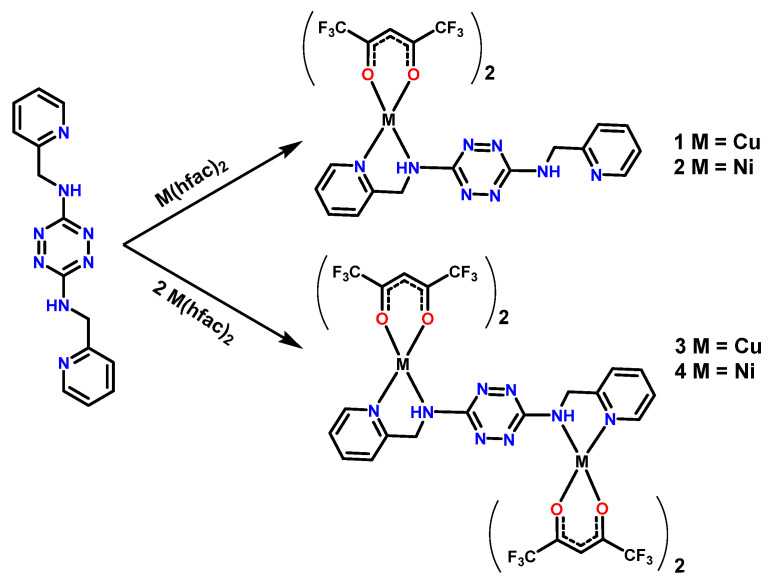
Synthesis of complexes **1**–**4**.

Since L is a ditopic ligand containing two chelating units, its reaction with metal centers could, in principle, lead to discrete mono- and binuclear metal complexes or coordination polymers, depending on the number of available coordination sites on the metal center. As mentioned above, in the course of a previous study [31] we indeed obtained a mononuclear cobalt(II) complex [Co(hfac)_2_(L)], where L acts as a monotopic chelating ligand, and a one-dimensional coordination polymer [Co(hfac)_2_(µ-L)]_n_, in which L connects cobalt(II) centers through coordination of the pyridines only. We extend herein our study towards the paramagnetic centers copper(II) and nickel(II) by the use of the neutral [M(hfac)_2_] precursors. First, reaction of L with one equivalent of [M(hfac)_2_] afforded the mononuclear complexes [Cu(hfac)_2_(L)] (**1**) and [Ni(hfac)_2_(L)] (**2**) (Scheme 2) where L acts as a monotopic bidentate ligand thanks to its N_Py_ and N_amine_ atoms. In both complexes, the ligand remains in its amino form and not amido, as already observed with the pica-TTZ-Cl ligand and [Cu(hfac)_2_] fragment [30].

This can be very likely explained by the lower acidity of the amino groups in L than in pica-TTZ-Cl, where the presence of the Cl substituent in the *para* position enhances the acidity of the NH group in the latter. On the other hand, reaction of L with an excess of [M(hfac)_2_] leads to the binuclear complexes [{Cu(hfac)_2_}_2_(µ-L)] (**3**) and [{Ni(hfac)_2_}_2_(µ-L)] (**4**) (Scheme 2) in which both picolylamino groups chelate the metal centers in a bidentate mode.

**Figure 1:**
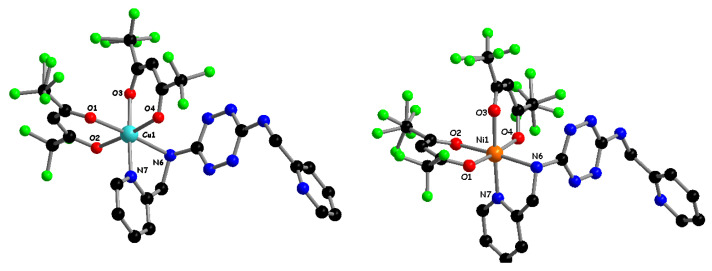
Crystal structures of **1** (**left**) and **2** (**right**) together with a partial atom numbering scheme. Some of the F atoms are disordered. Hydrogen atoms were omitted for clarity.

The mono- or binuclear nature of complexes **1**–**4** is definitely evidenced by single-crystal X-ray diffraction analysis. The best quality crystals for the copper(II) complexes **1** and **3** were obtained from methanol, while acetonitrile was successfully used for the nickel(II) complexes **2** and **4**. Complexes **1** and **2** are isostructural and crystallize in the monoclinic system, space group *P*2_1_/n, with one molecule of complex in the asymmetric unit. The ligand L coordinates to the metal ions through the nitrogen atoms of the picolylamine fragment, thus providing a slightly distorted MN_2_O_4_ octahedral environment (Figure 1, see Tables S1–S4 for bond lengths, bond angles and intermolecular C–H···F distances).

**Figure 2:**
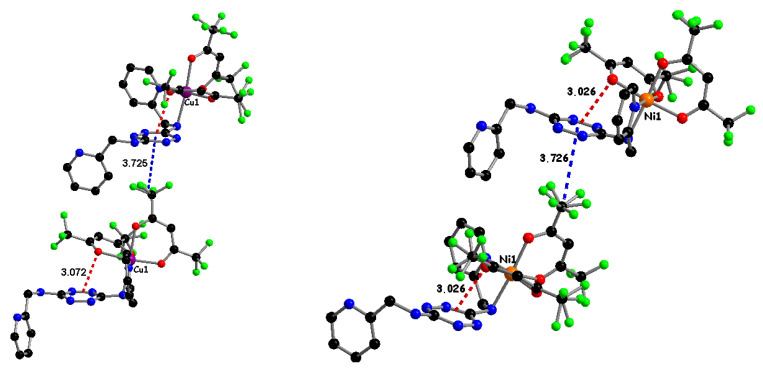
Anion–*π* interactions between TTZ and fluorine atoms (blue lines), and TTZ and oxygen atoms (red line) in **1** (**left**) and **2** (**right**). Hydrogen atoms were omitted for clarity.

The higher distortion of the octahedron at the copper(II) ion compared to nickel(II), observed in the corresponding structures **1** and **2**, is the consequence of the strong Jahn–Teller effect in the former. The Cu–O(N) bond distances varied from 1.940(2) to 2.318(3) Å, while for Ni–O(N) this range was much narrower, i.e., 2.016(2)–2.170(2) Å. The *cis* and *trans* O–Cu–O(N) bond angles spanned from 85.64(10) to 95.63(12)° and from 173.26(10) to 174.72(12)°, respectively. The corresponding angles for **2**, that is 85.53(8)–95.00(9)° and 174.78(8)–175.57(9)°, were similar to those in **1**. In the crystal packing, short intermolecular anion–*π* distances between tetrazine units and fluorine/oxygen atoms of the hfac ligands were observed (Figure 2).

Moreover, weak intermolecular C–H···F type interactions occurred between fluorine atoms and CH_2_ and Py protons (Figures S1 and S2, Tables S2 and S4). The closest metal···metal distances in the crystal packing amounted to 8.39 (**1**) and 8.55 Å (**2**).

**Figure 3:**
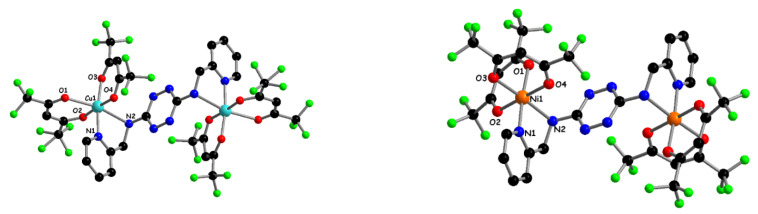
Crystal structures of **3** (**left**) and **4** (**right**) together with a partial atom numbering scheme. Hydrogen atoms and solvent molecules were omitted for clarity.

Although compounds **3** and **4** are not isostructural, they both crystallized in the triclinic system, space group *P*–1, with half a molecule of complex in the asymmetric unit, since the TTZ bridge was located on an inversion center (Figure 3, Tables S5 and S6).

Two solvent molecules of crystallization per molecule of complex were present in both crystal structures and therefore, the resulting formulas are [{Cu(hfac)_2_}_2_(µ-L)]·2CH_3_OH (**3**) and [{Ni(hfac)_2_}_2_(µ-L)]·2CH_3_CN (**4**). The coordination environment at each copper(II) ion in **3** is an elongated octahedron, due to the Jahn–Teller effect. The equatorial Cu–O(N) bond lengths varied from 1.960(3) to 1.995(4) Å, while the axial Cu–O1 and Cu–N2 distances were 2.270(4) and 2.498(5) Å, respectively. The O–Cu–O(N)*_cis_* bond angles varied from 86.95(14) to 92.29(14)°, while the O–Cu–O(N)*_trans_* angles spanned from 165.82(14) to 176.42(14)°. Each nickel(II) ion in **4** exhibits a distorted octahedral geometry (5 + 1) with a much narrower range of the equatorial Ni–O(N) bond lengths (2.0230(17)–2.0399(19) Å). The axial Ni–N2 distance was 2.1810(18) Å. The tight range of the values of the bond angles (the values of *cis*- and *trans*-angles being in the ranges 86.40(7)–97.17(7)° and 175.10(7)–176.76(6)°, respectively) indicates the lower distortion of the coordination octahedron compared to that at each copper center in **3**. The values of the metal···metal separation across the extended L bridge were 7.97 (**3**) and 7.82 Å (**4**). They were of the same order of magnitude as the shortest intermolecular metal···metal distances, i.e., 7.47 (**3**) and 7.83 Å (**4**).

**Figure 4:**
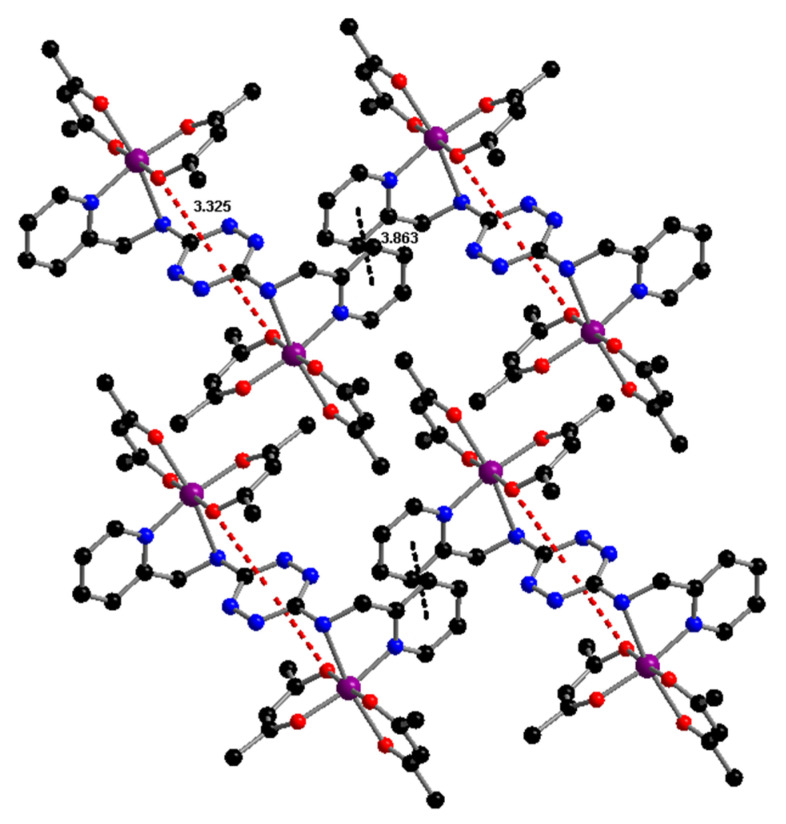
Intermolecular anion–π interactions between TTZ and oxygen atoms (red line) and π-π short distances between pyridyl rings (black line) in **3**. The hydrogen and fluorine atoms as well as the solvent molecules were omitted for clarity.

Intramolecular anion–π interactions between the tetrazine ring and oxygen atom (3.325 Å) (Figure S3), and intermolecular π···π relatively short distances between the pyridine rings (3.863 Å), were found in **3** (Figure 4), sustaining its supramolecular architecture. Similar intramolecular anion–π interactions (3.065 Å) existed in the structure of **4** (Figure S4).

### 2.2. Magnetic Properties of Complexes ***1**–**4***

**Figure 5:**
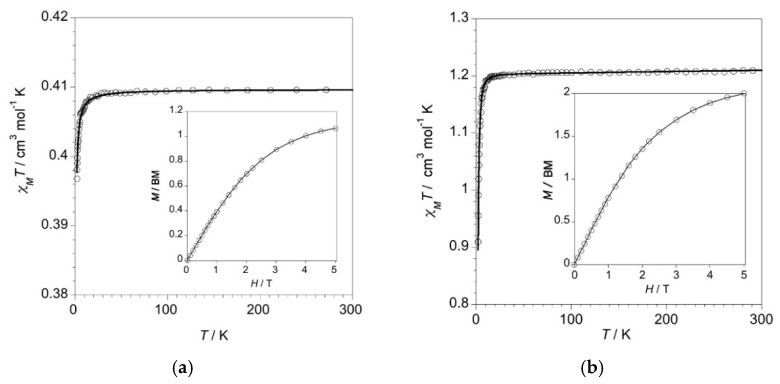
(**a**) Temperature dependence of the χ_M_*T* product for **1**: (o) experimental, (^__^) best-fit curve through Equation (1) (see text). (**b**) χ_M_*T* against T plot for **2**: (o) experimental, (^__^) best-fit curve through Equations (2–4) (see text). The inset shows the magnetization *versus H* plot for **2** at 2.0 K. The solid line is a guide for the eye.

The magnetic properties of **1** and **2** under the form of χ_M_*T* versus *T* plots [χ_M_ is the magnetic susceptibility per one copper(II) (**1**) or one nickel(II) (**2**) ions] are shown in Figure 5. The values of χ_M_*T* at room temperature were 0.41 (**1**) and 1.21 cm^3^ mol^−1^ K (**2**). They were as expected for one magnetically non-interacting doublet [χ_M_*T* = 0.41 cm^3^ mol^−1^ K with *S*_Cu_ = ½ and *g*_Cu_ = 2.10] (**1**) or triplet [χ_M_*T* = 1.21 cm^3^ mol^−1^ K with *S*_Ni_ = 1 and *g*_Ni_ = 2.20] (**2**) local spins. Upon cooling, these values remained constant until 22 (**1**) and 11 K (**2**) and they further decreased to 0.397 (**1**) and 0.91 cm^3^ mol^−1^ K (**2**) at 1.9 K. The small decrease of χ_M_*T* at very low temperatures for both compounds is attributed to weak intermolecular antiferromagnetic interactions (**1** and **2**) and/or zero-field splitting effects (**2**).

Let us analyze first the magnetic data of **1**. Having in mind its mononuclear nature and the above considerations, the magnetic data of this compound were treated through a Curie law for a copper(II) ion Equation (1)

χ_M_ = *Nß*^2^*g*^2^*S*(*S* + 1)/3*k*(*T* − θ) (1)

where *N*, *ß*, and *k* have their usual meanings, *S* = ½ and θ is a Curie–Weiss term that was introduced to take into account the intermolecular magnetic interactions. The least-squares best-fit parameters are *g* = 2.09(1) and θ = −0.06(1) K. This very weak magnetic coupling is in line with the great value of the shortest intermolecular copper(II)···copper(II) separation in **1** (ca. 9.5 Å). This is also reflected by the *M* vs. *H* plot for **1** at 2.0 K (see inset of Figure 5a), *M* tending to a quasi-saturation value close to 1.05 µ_B_ at 5 T (that is, the value calculated through *M*_sat_ = *g*_Cu_*S*_Cu_ with *g*_Cu_ = 2.09).

Keeping these results for **1** in mind, and given that the shortest intermolecular nickel···nickel separation in **2** is very large (ca. 9.4 Å), it is reasonable to assume that the observed decrease of χ_M_*T* for **2** at low temperatures was due to the zero-field splitting of the six-coordinate nickel(II) ion. The fact that the quasi-saturation value of *M* at 5 T for **2** (ca. 2.0 µ_B_) was somewhat below the expected value of *M*_S_ = *g*_Ni_*S*_Ni_ = 2.20 µ_B_ with S_Ni_ = 1 and *g*_Ni_ = 2.20) also supports the occurrence of a significant zfs (see inset of Figure 5b). Then, the magnetic susceptibility data of **2** were treated through Equations (2)–(4) [32]:

χ_M_ = (χ_M||_ + 2 χ_M__⊥_)/3 (2)

with

χ_M||_ = (2*Nß*^2^*g*^2^_||_/*kT*)[exp(−*D*/*kT*)/(1 + 2 exp(−*D*/*kT*] (3)

and

χ_M__⊥_ = (2*Nß*^2^*g*^2^_⊥_/*D*){[1 − exp(−*D*/*kT*)]/[1 + 2 exp(−*D*/*kT*)]} (4)

where *D* is the zero-field splitting and all other parameters have their usual meanings. Best-fit parameters are *g*_||_ = *g*_⊥_ = *g*_Ni_ = 2.20(1) and *D* = 4.48(2) cm^−1^. This value of *D* lies in the range of those reported for other six-coordinate nickel(II) complexes [33]. No ac signals were observed for **2,** either under zero or non-zero applied dc fields. Alternatively, the magnetic susceptibility data of **2** data were fitted by considering that only the intermolecular antiferromagnetic interactions would be responsible for the observed downturn of χ_M_*T* at low temperatures. The too-large value obtained of the Weiss parameter Weiss (*θ* = −0.545(2) K with *D* = 0 cm^−1^), and having in mind the above magneto-structural features, this last approach has fewer arguments for its use.

**Figure 6:**
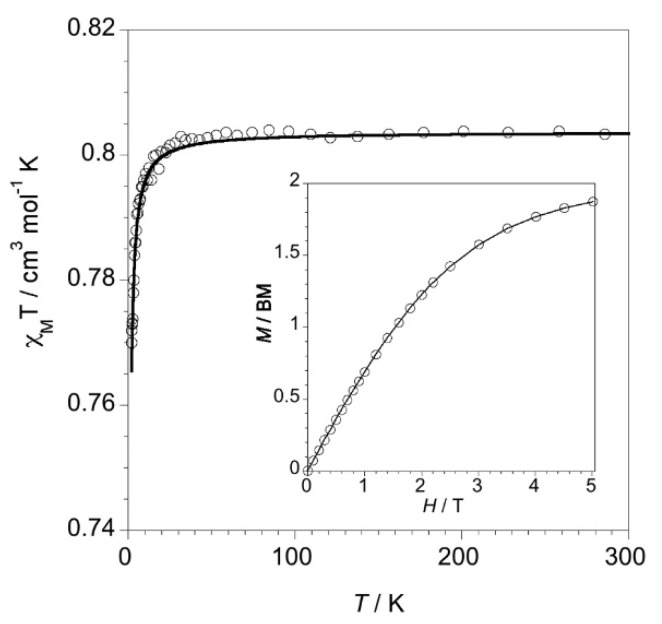
χ_M_*T* against *T* plot for **3**: (o) experimental, (^__^) best-fit curve through Equation (5) (see text). The inset shows the magnetization *versus H* plot for **3** at 2.0 K. The solid line is only an eye-guide.

The magnetic properties of **3** as χ_M_*T* against *T* plot [χ_M_ is the magnetic susceptibility per two copper(II) ions] are shown in Figure 6. At 300 K, χ_M_*T* for **3** was equal to 0.80 cm^3^ mol^−1^ K, a value which was as expected for two magnetically isolated spin doublets (ca. 0.82 cm^3^ mol^−1^ K with *g*_Cu_ = 2.10). This value was kept upon cooling until 40 K and it further decreased to 0.77 cm^3^ mol^−1^ K at 1.9 K. This small decrease in the low temperature region could be attributed to a very weak intramolecular antiferromagnetic coupling across the bis-bidentate L ligand (copper···copper separation of 7.97 Å), although the intermolecular contribution to the exchange coupling could be also envisaged because the shortest intermolecular metal···metal separation (ca. 7.47 Å) is comparable to the intramolecular one.

The analysis of the magnetic susceptibility data of **3** through the expression for a dicopper(II) complex is shown in Equation (5)

χ_M_ = (2*Nß*^2^*g*^2^/*kT*) [3 + exp(−*J*/*kT*)]^−1^ (5)

where *J* is the magnetic coupling after the spin Hamiltonian ***H*** = −*J**S***_Cu1_·***S***_Cu2_ and *g* is the average Landé factor. Best-fit parameters are *J* = −0.247(2) cm^−1^ and *g* = 2.07(1). This small coupling could be considered as the upper limit because of the possibility of intermolecular magnetic interactions. The relative orientation of the magnetic orbitals in **3** allow us to account for this weak intramolecular magnetic coupling. However, the very large value of the shortest intermolecular copper···copper separation in **2** (ca. 9.9 Å), allows us to rule out any significant intermolecular magnetic interaction. In fact, the unpaired electron at each copper(II) ion was mainly defined by a (dx^2^-y^2^)-type magnetic orbital which was mainly delocalized in the equatorial plane (N1O2O3O4 set of atoms, the *x* and *y* axis being roughly defined by the equatorial bonds). These magnetic orbitals were parallel to each other in the dicopper(II) unit and they were not properly oriented to interact efficiently in the region of the bridge. The spin density on the axial N2 and N2^i^ atoms [symmetry code: (i) = 1 − *x*, 2 − *y*, 1 − *z*] was predicted to be very small, and then, the overlap between the magnetic orbitals of the copper(II) ions of **3** through the extended bis-bidentate L ligand was expected to be very poor. According to the Kahn’s orbital model, the magnetic interaction in dicopper(II) complexes is proportional to the square of the overlap integral between the magnetic orbitals (the bielectronic exchange integrals being neglected) [34,35]. Then, a weak antiferromagnetic coupling was predicted for **3,** as observed. This is in agreement with the fact that *M* for **3** at 2.0 K tends to a value somewhat below 2.0 µ_B_ at 5 T (see inset of Figure 6).

**Figure 7:**
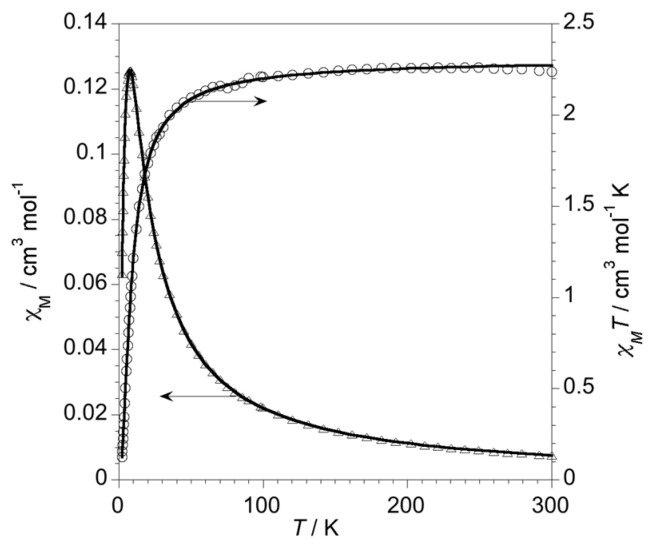
χ_M_*T* (o) and χ_M_ (∆) against *T* plots for **4**. The solid lines are the best-fit curves through Equation (6) (see text).

The magnetic properties of **4** in the form of both χ_M_*T* and χ_M_ vs. *T* plots (χ_M_ is the magnetic susceptibility per two nickel(II) ions) are shown in Figure 7. At 300 K, χ_M_*T* was equal to 2.28 cm^3^ mol^−1^ K, a value which is as expected for a pair of non-interacting spin triplets (χ_M_*T* = 2.28 cm^3^ mol^−1^ K with *S*_Ni_ = 1 and *g*_Ni_ = 2.15). This value continuously decreased upon cooling to reach a minimum value of 0.13 cm^3^ mol^−1^ K at 2.0 K. The magnetic susceptibility exhibited a maximum of 0.124 cm^3^ mol^−1^ K at 7.0 K. These features are typical of an overall relatively weak antiferromagnetic behavior between the two single-ion triplet states.

Given that the ground state for a six-coordinate nickel(II) ion is non-degenerate, the isotropic spin Hamiltonian ***H*** = −*J**S***_Ni1_·***S***_Ni2_ might be appropriate to analyze the intradimer magnetic coupling (*J*) of **4**. Nevertheless, this approach, neglecting the zfs (*D*), works well when a relatively strong antiferromagnetic coupling occurs. In the systems where a weak antiferromagnetic coupling takes place (as in **4**), or when the magnetic interaction is ferromagnetic, the effect of the *D* parameter of the six-coordinate nickel(II) ion has to be taken into account to describe properly the magnetic behavior at low temperatures [36]. This is why the magnetic susceptibility data of **4** were analyzed by the Hamiltonian of Equation (6)

***H*** = −*J**S***_Ni1_·***S***_Ni2_ + *D*(***S***^2^_z,Ni1_ + ***S***^2^_z,Ni2_) + *ßH*(*g*_Ni1_***S***_Ni1_ + *g*_Ni2_***S***_Ni2_) (6)

where the first term accounts for the intradimer exchange coupling, the second term corresponds to the axial zfs and the third one is the Zeeman interaction with *g*_Ni1_ = *g*_Ni2_ = *g*_Ni_. The best-fit parameters were *J* = −4.86(2) cm^−1^, *D* = 5.17(2) cm^−1^ and *g*_Ni_ = 2.15(1). The calculated χ_M_*T* and χ_M_ curves (solid lines in Figure 7) reproduced well the magnetic data in the whole temperature range explored.

Let us to finish with a brief comment about the intramolecular antiferromagnetic coupling in **4,** which was due to the spin polarization mechanism [37]. The two unpaired electrons at each six-coordinate nickel(II) ion in this complex are defined by d(x^2^−y^2^) and dz^2^ type orbitals, the former one being roughly localized in the equatorial plane (N1O1O2O4 set of atoms), whereas the latter one lies along the O3-Ni-N2 axis. As observed for **3**, the two d(x^2^ − y^2^) magnetic orbitals were parallel to each other and their orientation towards the bridge was not appropriate to delocalize any significant spin density on the skeleton of the bridge. This was not the case for the two dz^2^ orbitals, the spin density on the axial N2 site (and symmetry-related N2^i^ atom) being large. As the angle between the Ni–N2 vector and the mean plane of the tetrazine ring was close to 90° (ca. 109°), a significant π overlap between the two magnetic orbitals through the -N2-(CNNC)-N2^i^- bridging fragment was predicted. Because of the even number of atoms in this bridging skeleton, the alternating spin densities on it led to an antiferromagnetic coupling. An odd number of atoms in the π-pathway would result in a parallel alignment of the spins of the metal ions and then in a significant ferromagnetic interaction. The efficiency of this mechanism was nicely shown by Iwamura et al., in the context of organic radicals [38], and it was successfully extended to the domain of coordination chemistry with several paramagnetic transition metal ions [39,40,41,42,43,44,45,46,47,48,49,50,51,52,53,54,55,56].

## 3. Materials and Methods

Reactions were carried out under air, and using HPLC solvents. The IR spectra were recorded on ATR Bruker Vertex 70 spectrophotometer in the 4000−400 cm^−1^ range. MALDI-TOF MS spectra were recorded on Bruker Biflex-IIITM apparatus, equipped with a 337nm N_2_ laser. ESI-MS spectra were achieved on a Bruker MicrO-Tof-Q 2 spectrometer. Elemental analyses were recorded using a Flash 2000 Fisher Scientific Thermo Electron analyzer. Copper(II) and nickel(II) hexafluoroacetylacetonate hydrates were purchased from Sigma Aldrich and dehydrated under high vacuum prior to their use. The L ligand was prepared as previously described [31].

[Cu(hfac)_2_(L)] **1**: To a solution of **L** (0.02 g, 6.8 × 10^−5^ mol) in 10 mL of dichloromethane was added a solution of bis(hexafluoroacetylacetonato)copper(II)[Cu(hfac)_2_] (32.5 mg, 6.8 × 10^−5^ mol) in 10 mL of methanol. The mixture was stirred at room temperature for 1 h. The color changed from red to brown during the reaction. The resulting solution was left undisturbed for two days at room temperature. During this period, yellow-brown crystals (0.025 g, 47%), suitable for single-crystal X-ray analysis, were obtained. MALDI-TOF MS: *m/z* = 563.9 [Cu(hfac)(L)]^+^. ESI-MS: *m/z* = 563.9 [Cu(hfac)(**L**)]^+^. IR (ATR, cm^−1^): 3415 (w), 1643 (s), 1551 (m), 1252 (s), 1199 (s), 1135 (s), 928 (w), 793 (s), 669 (s), 583 (s), 516 (m). Elemental analysis calcd for C_24_H_16_F_12_N_8_O_4_Cu: C, 37.34; H, 2.09; N, 14.54%. Found: C, 37.55; H, 2.36; N 14.78%.

[Ni(hfac)_2_(L)] **2**: To a solution of L (0.02 g, 6.8 × 10^−5^ mol) in 10 mL of dichloromethane was added a solution of [Ni(hfac)_2_] (32.1 mg, 6.8 × 10^−5^ mol) in 10 mL of acetonitrile. The mixture was stirred at room temperature for one hour. As in **1**, the color of the solution changed from red to brown, evidencing the complex formation. The resulting solution was left undisturbed for one day at room temperature, yielding brown crystals (0.037 g, 70%) suitable for single-crystal X-ray analysis. ESI-MS: *m*/*z* = 766 [Ni(hfac)_2_(L)]^+^. IR (ATR, cm^−1^): 3219 (w), 1642 (s), 1482 (s), 1253 (s), 1189 (s), 1139 (s), 945 (w), 792 (s), 671 (s), 585 (s), 529 (m). Elemental analysis calcd for C_24_H_16_F_12_N_8_O_4_Ni: C, 37.57; H, 2.10; N, 14.60%. Found: C, 37.65; H, 2.44; N, 14.85%.

[{Cu(hfac)_2_}_2_(π-L)]·2CH_3_OH **3**: To a solution of L (0.02 g, 6.8 × 10^−5^ mol) in 10 mL of dichloromethane was added a solution of [Cu(hfac)_2_] (130 mg, 27.2 × 10^−5^ mol) in 20 mL of methanol. The mixture was stirred at 70 °C for one hour. During the reaction, the color changed from red to brown. The resulting solution was left undisturbed for one day at room temperature, yielding yellow-brown crystals (0.051 g, 57%) suitable for single-crystal X-ray analysis. MALDI-TOF MS: *m*/*z* = 1246 [{Cu(hfac)_2_}_2_(L)]^+^. IR (ATR, cm^−1^): 3415 (w), 1642 (s), 1552 (m), 1253 (s), 1199 (s), 1201 (s), 928 (w), 794 (s), 670 (s), 584 (s), 517 (m). Elemental analysis calcd for C_36_H_26_F_24_N_8_O_10_Cu_2_: C, 32.91; H, 1.99; N, 8.52%. Found: C, 33.30; H, 2.12; N, 8.67%.

[{Ni(hfac)_2_}_2_(π-L)]·2CH_3_CN **4**: To a solution of L (0.02 g, 6.8 × 10^−5^ mol) in 10 mL of dichloromethane was added a solution of [Ni(hfac)_2_] (96.5 mg, 20.4 × 10^−5^ mol) in 20 mL of acetonitrile. The mixture was stirred at 70 °C for one hour. During the reaction, the color changed from red to red-brown. The resulting solution was left undisturbed for one day at room temperature. Yellow-brown crystals (0.06 g, 67%) suitable for single-crystal X-ray analysis were collected after this period. MALDI-TOF MS: *m*/*z* = 1238 [{Ni(hfac)_2_}_2_(L)]^+^. IR (ATR, cm^−1^): 3418 (w), 1646 (s), 1548 (m), 1249 (s), 1203 (s), 1195 (s), 932 (w), 798 (s), 667 (s), 579 (s), 509 (m). Elemental analysis calcd for C_38_H_24_F_24_N_10_O_8_Ni_2_: C, 34.52; H, 1.83; N, 8.88%. Found: C, 34.89; H, 1.95; N 9.22%.

Variable temperature direct current (dc) magnetic susceptibility measurements (1.9–300 K) under applied dc magnetic fields of 5000 G (*T* ≥ 50 K) and 100 G (1.9 ≤ *T* < 50 K) and variable-field (0–5 T) magnetization measurements (2.0 K) on crushed crystals of **1**–**4** were carried out with a Quantum Design SQUID magnetometer. Alternating current (ac) magnetic susceptibility measurements at low temperatures (2.0–8.0 K) under different applied dc magnetic fields in the range 0–2500 G were performed for **2** by using a Quantum Design Physical Property Measurement System (PPMS). The magnetic susceptibility data of these compounds were corrected for the diamagnetism of the constituent atoms and the sample holder (a plastic bag).

X-ray single crystal diffraction data were collected on an Agilent Technologies SuperNova diffractometer equipped with AtlasCCD detector and micro-focus Cu-Kα radiation (λ = 1.54184 Å). The structures were solved by direct methods and refined on F^2^ by full matrix least-squares techniques using the SHELX97 (G.M. Sheldrick, 1998) package. All non-H atoms were refined anisotropically and multiscan empirical absorption was applied using the CrysAlisPro program (CrysAlisPro, AgilentTechnologies, V1.171.38.41r, 2015). The hydrogen atoms were included in the calculation without refinement. A summary of the crystallographic data and the structure refinement for single crystals of compounds **1**–**4** is given in Table 1.

Crystallographic data for the four structures have been deposited with the Cambridge Crystallographic Data Centre, deposition numbers CCDC 2070.862 (**1**), CCDC 2070.863 (**2**), CCDC 2070.864 (**3**), CCDC 2070.865 (**4**). These data can be obtained free of charge from CCDC, 12 Union road, Cambridge CB2 1EZ, UK (e-mail: deposit@ccdc.cam.ac.uk or http://www.ccdc.cam.ac.uk).

## 4. Conclusions

The versatility of 3,6-bis(picolylamino)-1,2,4,5-tetrazine (L) as a potentially monotopic and ditopic ligand has been exploited in this work through the preparation of mono- and binuclear copper(II) and nickel(II) complexes where the picolylamine fragments coordinate the metal centers in bidentate fashion. Their coordination sphere is completed by two hfac ligands, thus providing distorted octahedral MN_2_O_4_ coordination geometries in complexes **1**–**4**. The supramolecular solid state architecture of the complexes is sustained by intramolecular and intermolecular anion–π interactions between the tetrazine ring and oxygen and fluorine atoms, together with intermolecular π–π stacking. The magnetic properties of the four complexes have been investigated through static (dc) magnetic susceptibility measurements. Interestingly, the weak intramolecular antiferromagnetic interactions occurring in the binuclear Ni(II) complex **4** can be explained thanks to a spin polarization mechanism mediated by the tetrazine bridge. The involvement of the tetrazine (TTZ) ring in the magnetic coupling pathway is very interesting in view of the possibility to access the radical anion species of TTZ, which should strongly enhance the coupling between the two metal centers [17]. Besides, the selective preparation of mono- and binuclear species opens the way towards the controlled synthesis of heterobimetallic complexes.

## Figures and Tables

**Table 1:** Crystallographic data, details of data collection and structure refinement parameters for compounds **1**–**4**.

	1	2	3	4
Formula	C_24_H_16_F_12_N_8_O_4_Cu	C_24_H_16_F_12_N_8_O_4_Ni	C_36_H_26_F_24_N_8_O_10_Cu_2_	C_38_H_24_F_24_N_10_O_8_Ni_2_
*M* [g∙mol^−1^]	771.99	767.16	1313.75	1322.05
*T* [K]	89.9(3)	150.00(10)	150.00(10)	149.56(10)
Crystal system	monoclinic	monoclinic	triclinic	triclinic
Space group	*P*2_1_/*n*	*P*2_1_/*n*	*P*-1	*P*-1
*a* [Å]	9.479(3)	9.4370(10)	9.901(7)	9.2160(2)
*b* [Å]	14.191(5)	14.299(2)	11.416(7)	10.5636(3)
*c* [Å]	22.002(6)	22.162(4)	11.823(3)	13.4199(3)
*α* [°]	90	90	91.277(4)	89.369(2)
*β* [°]	98.581(2)	99.569(15)	96.619(5)	77.005(2)
*γ* [°]	90	90	100.342(5)	73.297(2)
*V* [Å^3^]	2926.5(16)	2948.9(7)	1304.6(13)	1217.30(5)
Z	4	4	1	1
*ρ*_calcd_ [g cm^−3^]	1.752	1.728	1.672	1.803
*μ* [mm^−1^]	2.216	2.103	2.343	2.388
Goodness-of-fit on *F*^2^	1.027	1.041	1.080	1.055
Final *R*_1_^a^/w*R*_2_^b^ [*I* > 2*σ*(*I*)]	0.0632/0.1576	0.0555/0.1457	0.0881/0.2453	0.0573/0.1524
*R*_1_^a^/w*R*_2_^b^ (all data)	0.0698/0.1635	0.0600/0.1498	0.0931/0.2525	0.0625/0.1589

^a^ R_1_ = ∑||F_o_|–|F_c_||/∑|F_o_|. ^b^ wR_2_ = [∑w(F_o_^2^ − F_c_^2^)^2^/∑w(F_o_^2^)^2^]^1/2^; w = 1/[σ^2^(F_0_^2^) + (aP)^2^ + bP] where P = [max(F_0_^2^, 0) + 2F_c_^2^]/3.

## Data Availability

Not applicable.

## Supplementary Materials

The following are available online, Figure S1: Intermolecular C–H·F hydrogen bonds in **1**, Figure S2: Intermolecular C–H F hydrogen bonds in **2**, Figure S3: Intermolecular anion-π interactions between TTZ and oxygen atoms (red line) in **3**. Hydrogen atoms and solvent molecules were omitted for clarity. Figure S4: Intermolecular anion-π interactions between TTZ and oxygen atoms (red line) in **4**. Hydrogen atoms and solvent molecules were omitted for clarity, Table S1: Bond lengths (Å) and bond angles (°) for **1**, Table S2: Hydrogen bonds parameters for **1**, Table S3: Bond lengths (Å) and bond angles (°) for **2**, Table S4: Hydrogen bonds parameters for **2**, Table S5: Bond lengths (Å) and bond angles (°) for **3**, Table S6: Bond lengths (Å) and bond angles (°) for **4**.

## Sample Availability

Samples of the compounds are not available from the authors.
