# Supplementary material for: Mono- and Binuclear Copper(II) and Nickel(II) Complexes with the 3,6-Bis(picolylamino)-1,2,4,5-Tetrazine Ligand
Source: Molecules. 2021 Apr 7;26(8):2122. doi: 10.3390/molecules26082122 (PMC8067877; doi:10.3390/molecules26082122)
Supplement: Supplementary file 1 [file molecules-26-02122-s001.pdf]

Supporting information for

Mono- and Binuclear Copper(II) and Nickel(II) Complexes with the 3,6-Bis(picolylamino)-1,2,4,5-Tetrazine Ligand

Oleh Stetsiuk [1], Abdelkrim El-Ghayoury [1], Francesc Lloret [2], Miguel Julve [2,*] and Narcis Avarvari [1,*]

[1] Univ Angers, CNRS, MOLTECH-Anjou, SFR MATRIX, F-49000 Angers, France; oleh.stetsiuk@polytechnique.edu (O.S.); abdelkrim.elghayoury@univ-angers.fr (A.E.-G.)

[2] Instituto de Ciencia Molecular (ICMol)/Departament de Química Inorgànica, Universitat de València, C/ Catedrático José Beltrán 2, 46980 Paterna (Valencia), Spain; Francisco.Lloret@uv.es (F.L.)

* Correspondence: Miguel.Julve@uv.es (M.J.); narcis.avarvari@univ-angers.fr (N.A.)

Single crystal X-ray structures

Compound [Cu(hfac)$_2$(L)] **1**

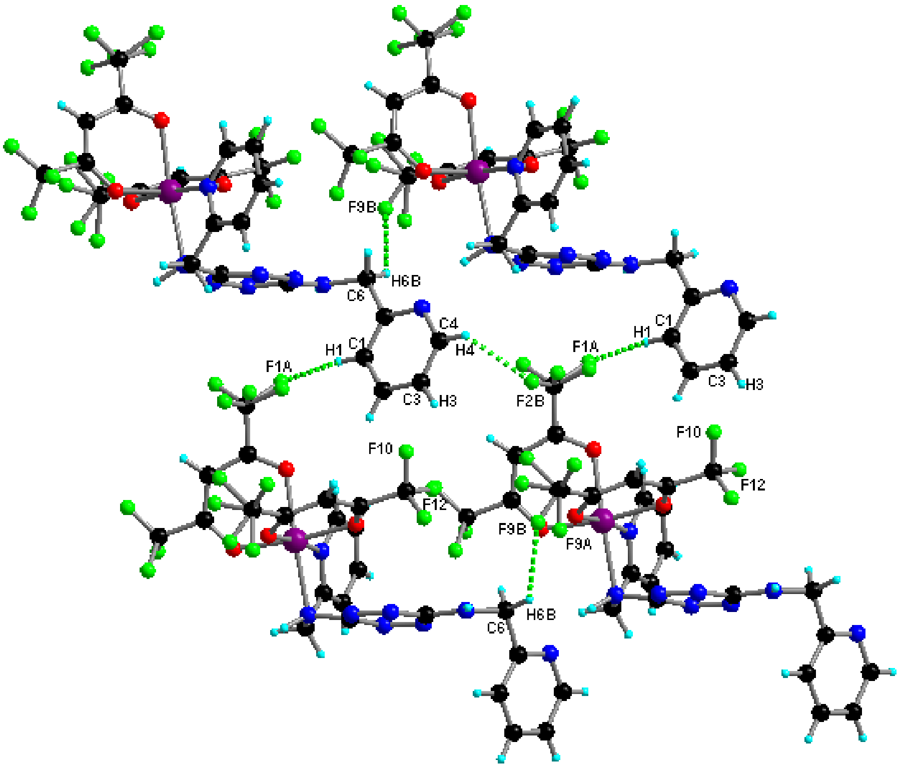

Figure S1. Intermolecular C–H···F hydrogen bonds in **1**.

Table S1. Bond lengths (Å) and bond angles (º) for **1**.

Distances	(Å)		
C1—C2	1.396(6)	C16—O1	1.246(4)
C2—C3	1.363(8)	C17—C18	1.388(5)
C3—C4	1.389(7)	C18—C19	1.512(7)
C4—N8	1.346(5)	C18—O2	1.263(4)
C5—C1	1.386(6)	C19—F4	1.334(6)
C5—N8	1.340(6)	C19—F5	1.336(5)
C6—C5	1.519(5)	C19—F6	1.339(5)
C6—N5	1.441(6)	C20—C21	1.537(5)
C7—N1	1.354(6)	C20—F7A	1.223(12)
C7—N3	1.347(5)	C20—F7B	1.482(16)
C7—N5	1.351(5)	C20—F8	1.311(4)
C8—N2	1.352(5)	C20—F9A	1.419(9)
C8—N4	1.322(6)	C20—F9B	1.227(12)
C8—N6	1.399(5)	C21—C22	1.383(5)
C9—N6	1.483(4)	C21—O3	1.260(4)
C10—C9	1.489(7)	C22—C23	1.404(5)
C10—N7	1.354(5)	C23—C24	1.533(5)
C11—C10	1.390(6)	C23—O4	1.250(4)
C12—C11	1.365(8)	C24—F10	1.325(4)
C13—C12	1.378(7)	C24—F11	1.319(4)
C14—C13	1.383(5)	C24—F12	1.324(4)
C14—N7	1.340(6)	N2—N1	1.312(5)
C15—C16	1.520(6)	N4—N3	1.330(5)
C15—F1A	1.421(8)	N6—Cu1	2.318(3)
C15—F1B	1.308(7)	N7—Cu1	1.973(3)
C15—F2A	1.253(9)	O1—Cu1	2.212(4)
C15—F2B	1.555(10)	O2—Cu1	2.040(4)
C15—F3	1.282(5)	O3—Cu1	1.940(2)
C16—C17	1.394(6)	O4—Cu1	2.088(2)
Angles	(˚)		
C1—C5—C6	122.6(4)	F8—C20—C21	113.3(3)
C2—C3—C4	119.0(4)	F8—C20—F7B	94.9(12)
C3—C2—C1	119.0(4)	F8—C20—F9A	98.5(6)
C5—C1—C2	118.6(5)	F9A—C20—C21	106.4(6)
C5—N8—C4	117.2(4)	F9A—C20—F7B	137.3(9)
C7—N5—C6	122.1(3)	F9A—F9B—C20	84.5(14)
C8—N4—N3	117.9(3)	F9A—F9B—F7A	137.8(14)
C8—N6—C9	115.6(3)	F9B—C20—C21	116.7(6)
C8—N6—Cu1	105.4(2)	F9B—C20—F7B	103.1(10)
C8—N6—H7	114.(3)	F9B—C20—F8	119.3(13)
C9—N6—Cu1	100.8(2)	F9B—C20—F9A	36.1(15)
C9—N6—H7	112.(3)	F9B—F9A—C20	59.4(11)
C10—N7—Cu1	117.9(3)	F10—C24—C23	112.4(3)
C11—C10—C9	123.3(4)	F11—C24—C23	111.2(3)
C11—C12—C13	119.7(4)	F11—C24—F10	106.4(3)
C12—C11—C10	120.2(4)	F11—C24—F12	107.7(3)
C12—C13—C14	118.4(5)	F12—C24—C23	112.4(3)
C14—N7—C10	119.7(3)	F12—C24—F10	106.3(3)
C14—N7—Cu1	122.4(2)	N1—N2—C8	116.8(4)
C16—C15—F2B	103.2(4)	N2—C8—N6	115.7(4)
C16—O1—Cu1	118.8(3)	N2—N1—C7	118.0(3)
C17—C16—C15	116.5(3)	N3—C7—N1	124.4(4)
C17—C18—C19	117.3(3)	N3—C7—N5	117.9(4)
C18—C17—C16	124.1(3)	N4—C8—N2	125.5(3)
C18—O2—Cu1	124.2(3)	N4—C8—N6	118.7(3)
C20—F7A—F9B	54.5(5)	N4—N3—C7	116.8(4)
C20—F9B—F7A	54.3(12)	N5—C6—C5	114.4(4)
C21—C22—C23	122.3(3)	N5—C7—N1	117.7(3)
C21—O3—Cu1	124.7(2)	N6—C9—C10	112.2(3)

C22—C21—C20	118.4(3)	N7—C10—C11	119.9(4)
C22—C23—C24	116.8(3)	N7—C10—C9	116.8(3)
C23—O4—Cu1	121.7(2)	N7—C14—C13	122.1(4)
F1A—C15—C16	114.0(4)	N7—Cu1—N6	79.11(13)
F1A—C15—F2B	132.0(5)	N7—Cu1—O1	95.74(12)
F1A—F1B—C15	70.0(5)	N7—Cu1—O2	90.83(13)
F1A—F1B—F2A	123.3(7)	N7—Cu1—O4	91.11(12)
F1B—C15—C16	114.4(4)	N8—C4—C3	123.2(5)
F1B—C15—F1A	50.2(4)	N8—C5—C1	123.1(4)
F1B—C15—F2B	87.7(5)	N8—C5—C6	114.3(4)
F1B—F1A—C15	59.9(5)	O1—C16—C15	113.7(4)
F1B—F2A—C15	65.1(5)	O1—C16—C17	129.8(4)
F2A—C15—C16	116.9(5)	F7A—C20—F9B	71.2(14)
F2A—C15—F1A	100.4(5)	F7A—F7B—C20	55.6(9)
F2A—C15—F1B	54.6(5)	F7B—C20—C21	105.0(8)
F2A—C15—F2B	33.2(4)	F7B—F7A—C20	92.0(13)
F2A—C15—F3	109.5(5)	F7B—F7A—F9B	144.9(15)
F2A—F1B—C15	60.3(5)	O1—Cu1—N6	174.72(12)
F2A—F2B—C15	53.6(8)	O2—C18—C17	128.7(4)
F2B—F2A—C15	93.2(9)	O2—C18—C19	113.9(4)
F2B—F2A—F1B	157.5(12)	O2—Cu1—N6	93.47(12)
F3—C15—C16	117.0(3)	O2—Cu1—O1	87.74(11)
F3—C15—F1A	96.1(5)	O2—Cu1—O4	173.26(10)
F3—C15—F1B	127.0(5)	O3—C21—C20	112.6(3)
F3—C15—F2B	92.9(4)	O3—C21—C22	129.0(3)
F4—C19—C18	111.9(4)	O3—Cu1—N6	95.63(12)
F4—C19—F5	105.9(5)	O3—Cu1—N7	174.55(13)
F4—C19—F6	106.3(3)	O3—Cu1—O1	89.54(11)
F5—C19—C18	114.5(3)	O3—Cu1—O2	88.05(12)
F5—C19—F6	107.2(3)	O3—Cu1—O4	90.60(9)
F6—C19—C18	110.6(4)	O4—C23—C22	128.0(3)
F7A—C20—C21	114.2(5)	O4—C23—C24	115.2(3)
F7A—C20—F7B	32.4(8)	O4—Cu1—N6	93.24(11)
F7A—C20—F8	115.7(8)	O4—Cu1—O1	85.64(10)
F7A—C20—F9A	106.8(6)	Cu1—N6—H7	107.(3)

Table S2. Hydrogen bonds parameters for **1**.

**d(A···H), Å**	**d(A···D) , Å**	**∠ (A-H-D), °**
F(9B)···H(6B) = 2.59	F(9B)···C(6) = 3.27	128
F(1A)···H(1) = 2.22	F(1A)···C(1) = 2.75	115
F(2B)···H(4) = 2.49	F(2B)···C(4) = 3.10	124

Compound [Ni(hfac)$_2$(L)] **2**

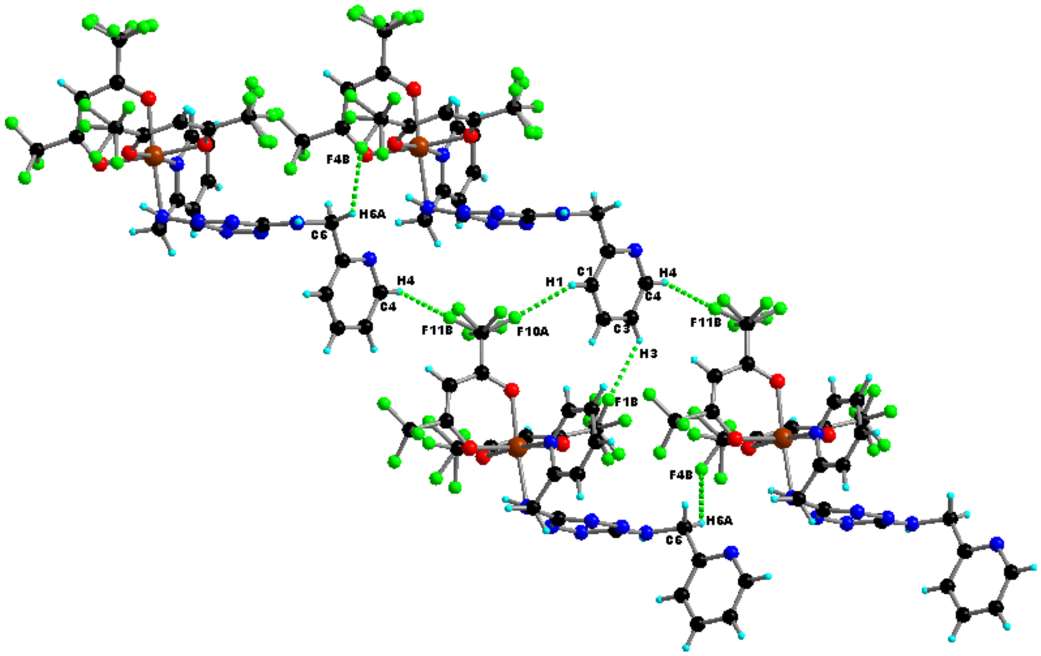

Figure S2. Intermolecular C–H···F hydrogen bonds in **2**.

Table S3. Bond lengths (Å) and bond angles (º) for **2**.

Distances	(Å)		
C1—C2	1.389(5)	C17—C18	1.397(4)
C1—C5	1.380(5)	C18—C19	1.534(5)
C2—C3	1.366(6)	C18—O3	1.248(4)
C3—C4	1.379(6)	C19—F4A	1.435(11)
C4—N8	1.342(4)	C19—F4B	1.296(8)
C5—C6	1.515(4)	C19—F5	1.291(4)
C5—N8	1.335(4)	C19—F6A	1.146(12)
C6—N5	1.454(4)	C19—F6B	1.434(10)
C7—N1	1.363(4)	C20—C21	1.538(4)
C7—N3	1.343(4)	C20—F7	1.305(4)
C7—N5	1.342(4)	C20—F8	1.320(5)
C8—N2	1.347(4)	C20—F9	1.313(4)
C8—N4	1.323(4)	C21—C22	1.387(4)
C8—N6	1.412(3)	C21—O1	1.259(3)
C9—C10	1.500(5)	C22—C23	1.391(4)
C9—N6	1.482(3)	C23—C24	1.525(4)
C10—C11	1.386(4)	C23—O2	1.243(3)
C10—N7	1.346(4)	C24—F10A	1.290(5)
C11—C12	1.374(5)	C24—F10B	1.38(3)
C12—C13	1.384(5)	C24—F11A	1.321(4)
C13—C14	1.377(4)	C24—F11B	1.254(16)
C14—N7	1.346(4)	C24—F12A	1.347(5)
C15—C16	1.529(4)	C24—F12B	1.214(11)
C15—F1A	1.360(5)	N1—N2	1.313(3)
C15—F1B	1.197(15)	N3—N4	1.331(3)
C15—F2A	1.286(5)	N6—Ni1	2.170(2)
C15—F2B	1.378(9)	N7—Ni1	2.030(2)
C15—F3A	1.313(5)	O1—Ni1	2.0368(19)
C15—F3B	1.193(14)	O2—Ni1	2.043(2)
C16—C17	1.392(4)	O3—Ni1	2.016(2)
C16—O4	1.261(4)	O4—Ni1	2.0428(19)
**Angles**	**(˚)**		
C1—C5—C6	123.5(3)	F6A—C19—F6B	31.7(8)
C2—C3—C4	118.1(3)	F6A—F6B—C19	52.6(13)
C3—C2—C1	118.8(3)	F6B—C19—C18	109.7(5)
C5—C1—C2	119.6(3)	F6B—C19—F4A	132.4(6)
C5—N8—C4	117.4(3)	F6B—F6A—C19	96.(2)
C7—N5—C6	121.7(2)	F6B—F6A—F4B	151.(2)
C8—N4—N3	117.6(2)	F7—C20—C21	112.7(3)
C8—N6—C9	114.9(2)	F7—C20—F8	105.7(3)
C8—N6—Ni1	107.67(16)	F7—C20—F9	107.1(3)
C9—N6—Ni1	103.84(17)	F8—C20—C21	109.5(3)
C10—N7—Ni1	115.5(2)	F9—C20—C21	113.2(2)
C11—C10—C9	123.4(3)	F9—C20—F8	108.3(3)
C11—C12—C13	119.4(3)	F10A—C24—C23	113.7(3)
C12—C11—C10	119.6(3)	F10A—C24—F10B	51.3(9)
C14—C13—C12	118.3(3)	F10A—C24—F11A	109.3(4)
C14—N7—C10	119.0(2)	F10A—C24—F12A	105.2(4)
C14—N7—Ni1	125.56(19)	F10A—F10B—C24	60.5(14)
C15—F1A—F3B	50.3(6)	F10A—F10B—F11A	103.7(19)
C15—F1B—F2A	62.2(6)	F10A—F12B—C24	68.1(7)
C15—F2A—F1B	55.4(8)	F10A—F12B—F12A	133.6(11)
C15—F2B—F3A	50.4(4)	F10B—C24—C23	103.7(7)
C15—F3A—F2B	54.0(4)	F10B—F10A—C24	68.2(9)
C15—F3B—F1A	61.3(5)	F11A—C24—C23	114.6(3)
C16—C17—C18	121.8(3)	F11A—C24—F10B	69.3(8)
C16—O4—Ni1	122.60(18)	F11A—C24—F12A	104.3(3)
C17—C16—C15	116.9(3)	F11A—F11B—C24	74.7(14)
C17—C18—C19	117.5(3)	F11A—F11B—F12A	130.8(17)

C18—O3—Ni1	123.46(19)	F11B—C24—C23	115.7(7)
C19—F4B—F6A	51.1(7)	F11B—C24—F10A	129.7(7)
C19—F6A—F4B	61.7(6)	F11B—C24—F10B	106.8(11)
C21—C22—C23	123.1(3)	F11B—C24—F11A	39.1(8)
C21—O1—Ni1	121.70(17)	F11B—C24—F12A	67.0(9)
C22—C21—C20	117. 5(2)	F11B—F11A—C24	66.3(10)
C22—C23—C24	117.7(2)	F11B—F11A—F10B	121.0(15)
C23—O2—Ni1	123.10(18)	F12A—C24—C23	108.9(3)
C24—F10B—F11A	53.6(7)	F12A—C24—F10B	146.0(8)
C24—F11A—F10B	57.1(10)	F12A—F12B—C24	67.9(8)
C24—F11B—F12A	59.6(6)	F12B—C24—C23	116.8(7)
C24—F12A—F11B	53.4(8)	F12B—C24—F10A	51.1(7)
F1A—C15—C16	113.2(3)	F12B—C24—F10B	101.2(11)
F1A—C15—F2B	133.3(5)	F12B—C24—F11A	128.5(7)
F1A—F1B—C15	75.1(13)	F12B—C24—F11B	110.7(11)
F1A—F1B—F2A	125.8(15)	F12B—C24—F12A	55.5(8)
F1B—C15—C16	108.0(5)	F12B—F10A—C24	60.8(7)
F1B—C15—F1A	46.6(8)	F12B—F10A—F10B	126.9(13)
F1B—C15—F2A	62.4(7)	F12B—F12A—C24	56.6(5)
F1B—C15—F2B	103.3(8)	F12B—F12A—F11B	100.4(10)
F1B—C15—F3A	138.8(6)	N1—N2—C8	116.7(2)
F1B—F1A—C15	58.2(7)	N2—C8—N6	116.2(2)
F1B—F1A—F3B	105.9(11)	N2—N1—C7	117.6(2)
F2A—C15—C16	114.9(3)	N3—C7—N1	124.2(2)
F2A—C15—F1A	102.3(4)	N4—C8—N2	125.8(2)
F2A—C15—F2B	41.6(5)	N4—C8—N6	118.0(2)
F2A—C15—F3A	110.7(4)	N4—N3—C7	116.9(2)
F2A—F2B—C15	64.0(6)	N5—C6—C5	114.1(3)
F2A—F2B—F3A	107.7(9)	N5—C7—N1	117.5(2)
F2B—C15—C16	110.1(5)	N5—C7—N3	118.2(2)
F2B—F2A—C15	74.4(6)	N6—C9—C10	111.8(2)
F2B—F2A—F1B	128.8(11)	N7—C10—C11	121.0(3)
F3A—C15—C16	111.0(3)	N7—C10—C9	115.6(2)
F3A—C15—F1A	104.0(4)	N7—C14—C13	122.6(3)
F3A—C15—F2B	75.6(7)	N7—Ni1—N6	80.38(9)
F3A—F3B—C15	79.1(14)	N7—Ni1—O1	91.90(9)
F3A—F3B—F1A	135.8(17)	N7—Ni1—O2	95.38(9)
F3B—C15—C16	114.0(6)	N7—Ni1—O4	92.33(9)
F3B—C15—F1A	68.3(7)	N8—C4—C3	124.1(3)
F3B—C15—F1B	112.1(9)	N8—C5—C1	122.0(3)
F3B—C15—F2A	129.6(7)	N8—C5—C6	114.5(3)
F3B—C15—F2B	108.8(9)	O1—C21—C20	112.6(2)
F3B—C15—F3A	37.7(7)	O1—C21—C22	129.9(3)
F3B—F3A—C15	63.2(10)	O1—Ni1—N6	90.38(8)
F3B—F3A—F2B	111.5(12)	O1—Ni1—O2	91.04(8)
F4A—C19—C18	103.4(4)	O1—Ni1—O4	174.78(8)
F4A—F4B—C19	80.8(11)	O2—C23—C22	128.5(3)
F4A—F4B—F6A	131.9(14)	O2—C23—C24	113.9(2)
F4B—C19—C18	116.2(4)	O2—Ni1—N6	175.57(9)
F4B—C19—F4A	36.1(6)	O2—Ni1—O4	85.53(8)
F4B—C19—F6B	97.3(7)	O3—C18—C17	128.7(3)
F4B—F4A—C19	63.0(9)	O3—C18—C19	113.8(3)
F5—C19—C18	114.8(3)	O3—Ni1—N6	95.00(9)
F5—C19—F4A	95.7(5)	O3—Ni1—N7	175.10(9)
F5—C19—F4B	115.4(6)	O3—Ni1—O1	86.48(8)
F5—C19—F6B	100.3(5)	O3—Ni1—O2	89.28(8)
F6A—C19—C18	113.8(6)	O3—Ni1—O4	89.54(8)
F6A—C19—F4A	103.3(8)	O4—C16—C15	114.2(3)
F6A—C19—F4B	67.2(8)	O4—C16—C17	128.8(3)
F6A—C19—F5	121.3(7)	O4—Ni1—N6	93.33(8)

Table S4. Hydrogen bonds parameters for **2**.

**d(A···H), Å**	**d(A···D) , Å**	**∠ (A-H-D), °**
F(4B)···H(6A) = 2.62	F(4B)···C(6) = 3.33	130
F(11B)···H(4) = 2.22	F(11B)···C(4) = 2.69	97
F(10A)···H(1) = 2.46	F(10A)···C(1) = 2.94	112
F(1B)···H(3) = 2.38	F(1B)···C(3) = 3.06	130

Compound $[\{Cu(hfac)_2\}_2(\mu\text{-}L)]\cdot 2CH_3OH$ **3**

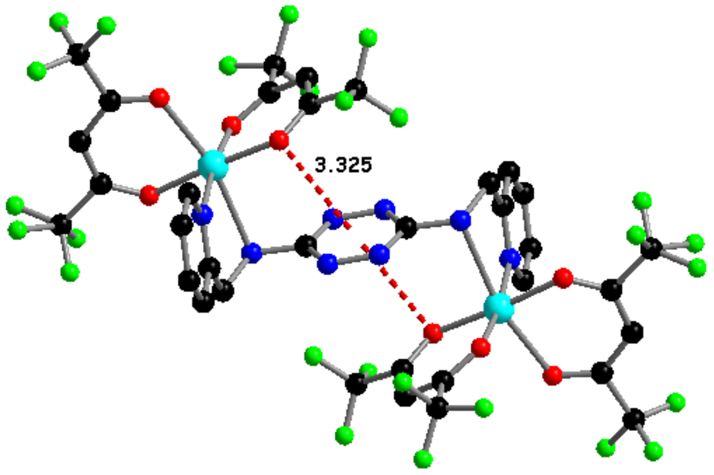

Figure S3. Intermolecular anion-π interactions between TTZ and oxygen atoms (red line) in **3**. Hydrogen atoms and solvent molecules were omitted for clarity.

Table S5. Bond lengths (Å) and bond angles (º) for **3**.

Distances	(Å)		
C1—N3	1.328(6)	C12—F6	1.348(15)
C1—N4	1.353(6)	C12—F4A	1.408(17)
C1—N2	1.378(6)	C13—F9	1.293(7)
C2—N2	1.452(6)	C13—F7	1.327(9)
C2—C3	1.512(7)	C13—F8	1.332(8)
C3—N1	1.343(6)	C13—C14	1.532(7)
C3—C4	1.391(7)	C14—O3	1.260(7)
C4—C5	1.374(9)	C14—C15	1.385(8)
C5—C6	1.394(9)	C15—C16	1.368(8)
C6—C7	1.364(8)	C16—O4	1.261(6)
C7—N1	1.347(7)	C16—C17	1.528(8)
C8—F2	1.296(9)	C17—F12	1.307(7)
C8—F1	1.298(9)	C17—F10	1.320(6)
C8—F3	1.319(8)	C17—F11	1.339(8)
C8—C9	1.539(8)	C18—O5	1.386(12)
C9—O1	1.225(6)	N1—Cu1	1.995(4)
C9—C10	1.399(9)	N3—N4	1.320(6)
C10—C11	1.361(9)	O1—Cu1	2.270(4)
C11—O2	1.255(7)	O2—Cu1	1.962(4)
C11—C12	1.539(10)	O3—Cu1	1.961(4)
C12—F4B	1.286(10)	O4—Cu1	1.960(3)
C12—F5	1.296(13)		
**Angles**	**(˚)**		
C1—N2—C2	119.0(4)	F7—C13—C14	112.1(5)
C3—N1—C7	119.8(4)	F7—C13—F8	104.9(5)
C3—N1—Cu1	120.7(3)	F8—C13—C14	112.2(5)
C4—C3—C2	121.1(5)	F9—C13—C14	110.8(5)
C4—C5—C6	118.9(5)	F9—C13—F7	108.0(7)
C5—C4—C3	120.1(5)	F9—C13—F8	108.6(6)
C7—C6—C5	118.6(5)	F10—C17—C16	112.4(5)
C7—N1—Cu1	119.5(3)	F10—C17—F11	105.4(5)
C9—O1—Cu1	121.1(3)	F11—C17—C16	112.5(5)
C10—C11—C12	117.4(6)	F12—C17—C16	110.9(5)
C10—C9—C8	116.0(5)	F12—C17—F10	108.8(5)
C11—C10—C9	124.5(5)	F12—C17—F11	106.6(5)

C11—O2—Cu1	127.6(4)	N1—C3—C2	118.7(4)
C12—F4A—F5	47.7(8)	N1—C3—C4	120.2(5)
C12—F4B—F4A	63.5(8)	N1—C7—C6	122.4(5)
C12—F5—F4A	53.5(9)	N1—Cu1—O1	96.39(15)
C14—O3—Cu1	124.8(4)	N2—C2—C3	113.6(4)
C15—C14—C13	117.6(5)	N3—C1—N2	118.2(4)
C15—C16—C17	118.4(5)	N3—C1—N4	125.3(4)
C16—C15—C14	121.7(5)	N3—N4—C1	116.7(4)
C16—O4—Cu1	124.9(3)	N4—C1—N2	116.4(4)
F1—C8—C9	111.6(6)	N4—N3—C1	117.9(4)
F1—C8—F3	105.7(6)	O1—C9—C10	128.2(5)
F2—C8—C9	113.2(5)	O1—C9—C8	115.9(5)
F2—C8—F1	108.6(8)	O2—C11—C10	129.4(6)
F2—C8—F3	104.9(7)	O2—C11—C12	113.2(6)
F3—C8—C9	112.4(5)	O2—Cu1—N1	90.30(16)
F4A—C12—C11	107.9(10)	O2—Cu1—O1	86.95(14)
F4B—C12—C11	115.6(7)	O3—C14—C13	114.4(5)
F4B—C12—F4A	61.6(11)	O3—C14—C15	128.0(5)
F4B—C12—F5	126.2(9)	O3—Cu1—N1	171.31(15)
F4B—C12—F6	95.(1)	O3—Cu1—O1	92.29(14)
F4B—F4A—C12	54.9(10)	O3—Cu1—O2	90.43(16)
F4B—F4A—F5	95.3(12)	O4—C16—C15	128.2(5)
F5—C12—C11	110.2(9)	O4—C16—C17	113.3(5)
F5—C12—F4A	78.8(13)	O4—Cu1—N1	87.66(15)
F5—C12—F6	94.8(9)	O4—Cu1—O1	96.19(13)
F6—C12—C11	110.0(8)	O4—Cu1—O2	176.42(14)
F6—C12—F4A	141.4(10)	O4—Cu1—O3	91.16(15)

Compound $[\{Ni(hfac)_2\}_2(\mu\text{-}L)]\cdot 2CH_3CN$ **4**

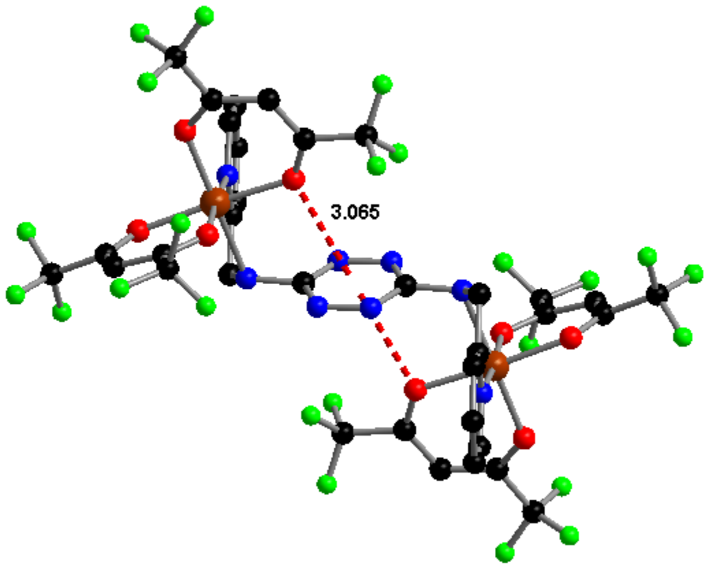

Figure S4. Intermolecular anion-π interactions between TTZ and oxygen atoms (red line) in **4**. Hydrogen atoms and solvent molecules were omitted for clarity.

Table S6. Bond lengths (Å) and bond angles (º) for **4**.

Distances	(Å)		
C1—N3	1.339(3)	C12—F5	1.338(3)
C1—N4	1.339(3)	C13—F8	1.306(3)
C1—N2	1.403(3)	C13—F7	1.317(3)
C2—N2	1.483(3)	C13—F9	1.330(3)
C2—C3	1.514(3)	C13—C14	1.541(3)
C3—N1	1.338(3)	C14—O3	1.251(3)
C3—C4	1.389(3)	C14—C15	1.392(3)
C4—C5	1.384(4)	C15—C16	1.393(3)
C5—C6	1.389(4)	C16—O4	1.250(3)
C6—C7	1.379(3)	C16—C17	1.534(3)
C7—N1	1.348(3)	C17—F11	1.291(4)
C8—F3	1.319(3)	C17—F10	1.318(3)
C8—F1	1.328(3)	C17—F12	1.322(3)
C8—F2	1.340(3)	C18—N5	1.136(4)
C8—C9	1.535(3)	C18—C19	1.447(4)
C9—O1	1.251(3)	N1—Ni1	2.0399(19)
C9—C10	1.396(3)	N2—Ni1	2.1810(18)
C10—C11	1.390(3)	N3—N4	1.325(3)
C11—O2	1.254(3)	O1—Ni1	2.0230(17)
C11—C12	1.532(3)	O2—Ni1	2.0373(15)
C12—F6	1.320(3)	O3—Ni1	2.0309(16)
C12—F4	1.331(3)	O4—Ni1	2.0320(15)
Angles	(˚)		
C1—N2—C2	116.84(18)	F10—C17—C16	113.1(2)
C1—N2—Ni1	109.10(13)	F10—C17—F12	105.7(2)
C2—N2—Ni1	105.01(13)	F11—C17—C16	111.0(2)
C3—N1—C7	119.11(19)	F11—C17—F10	108.0(3)
C3—N1—Ni1	115.77(14)	F11—C17—F12	107.1(3)
C4—C3—C2	121.8(2)	F12—C17—C16	111.7(2)
C4—C5—C6	119.2(2)	N1—C3—C2	116.35(19)
C5—C4—C3	118.9(2)	N1—C3—C4	121.8(2)
C7—C6—C5	118.7(2)	N1—C7—C6	122.2(2)

C7—N1—Ni1	125.08(16)	N1—Ni1—N2	79.67(7)
C9—O1—Ni1	125.16(15)	N2—C2—C3	111.49(17)
C10—C11—C12	117.6(2)	N3—C1—N2	115.79(19)
C10—C9—C8	118.2(2)	N3—C1—N4	126.6(2)
C11—C10—C9	122.2(2)	N3—N4—C1	116.78(18)
C11—O2—Ni1	124.57(14)	N4—C1—N2	17.49(18)
C14—C15—C16	122.3(2)	N4—N3—C1	116.62(19)
C14—O3—Ni1	123.24(15)	N5—C18—C19	179.0(3)
C15—C14—C13	117.7(2)	O1—C9—C10	128.4(2)
C15—C16—C17	117.9(2)	O1—C9—C8	113.4(2)
C16—O4—Ni1	124.22(14)	O1—Ni1—N1	175.10(7)
F1—C8—C9	110.1(2)	O1—Ni1—N2	95.60(7)
F1—C8—F2	107.4(2)	O1—Ni1—O2	89.94(7)
F2—C8—C9	112.4(2)	O1—Ni1—O3	87.59(7)
F3—C8—C9	111.8(2)	O1—Ni1—O4	88.33(7)
F3—C8—F1	107.7(2)	O2—C11—C10	129.0(2)
F3—C8—F2	107.2(2)	O2—C11—C12	113.36(19)
F4—C12—C11	113.8(2)	O2—Ni1—N1	91.02(7)
F4—C12—F5	106.1(2)	O2—Ni1—N2	86.40(7)
F5—C12—C11	110.8(2)	O3—C14—C13	113.1(2)
F6—C12—C11	110.69(19)	O3—C14—C15	129.1(2)
F6—C12—F4	108.1(2)	O3—Ni1—N1	97.17(7)
F6—C12—F5	106.9(2)	O3—Ni1—N2	176.62(7)
F7—C13—C14	111.9(2)	O3—Ni1—O2	92.49(6)
F7—C13—F9	105.8(2)	O3—Ni1—O4	90.18(6)
F8—C13—C14	109.6(2)	O4—C16—C15	128.6(2)
F8—C13—F7	107.5(2)	O4—C16—C17	113.43(19)
F8—C13—F9	108.4(2)	O4—Ni1—N1	90.48(7)
F9—C13—C14	113.4(2)	O4—Ni1—N2	91.04(6)
		O4—Ni1—O2	176.76(6)
